# Association between nutritional status and gait performance in Alzheimer's disease

**DOI:** 10.1111/cns.14502

**Published:** 2023-11-10

**Authors:** Mingyue He, Tenghong Lian, Peng Guo, Yanan Zhang, Yue Huang, Jing Qi, Jinghui Li, Huiying Guan, Dongmei Luo, Zhan Liu, Weijia Zhang, Zijing Zheng, Hao Yue, Jing Li, Wenjing Zhang, Ruidan Wang, Fan Zhang, Xiaomin Wang, Wei Zhang

**Affiliations:** ^1^ Department of Neurology, Beijing Tiantan Hospital Capital Medical University Beijing China; ^2^ Center for Cognitive Neurology, Department of Neurology, Beijing Tiantan Hospital Capital Medical University Beijing China; ^3^ Department of Blood Transfusion, Beijing Tiantan Hospital Capital Medical University Beijing China; ^4^ China National Clinical Research Center for Neurological Diseases, Beijing Tiantan Hospital Capital Medical University Beijing China; ^5^ Department of Pharmacology, School of Medical Sciences, Faculty of Medicine & Health UNSW Sydney Sydney New South Wales Australia; ^6^ Department of Physiology Capital Medical University Beijing China; ^7^ Center of Parkinson's Disease Beijing Institute for Brain Disorders Beijing China; ^8^ Beijing Key Laboratory on Parkinson Disease Beijing China

**Keywords:** Alzheimer's disease, dementia, gait performance, gait variability, mild cognitive impairment, nutritional status

## Abstract

**Aims:**

This study aimed to comprehensively explore the nutrition and gait of AD patients at different stages and the relationship between them.

**Methods:**

A total of 85 AD patients were consecutively enrolled in this cross‐sectional study and divided into the mild cognitive impairment (MCI) due to AD (AD‐MCI) and the dementia due to AD (AD‐D) groups. Demographic information, nutritional status, and gait performance were compared between the two groups, and the correlation between nutritional status and gait performance was subsequently analyzed by Pearson and Spearman correlation analyses.

**Results:**

The AD‐D group had lower scores on Mini‐Nutritional Assessment (MNA) and MNA^m^ scales, lower levels of urea nitrogen, folic acid, and vitamin B_12_ in blood, and higher homocysteine level than those in the AD‐MCI group (all *p* < 0.05). The AD‐D group had slower step speed, shorter step length, and shorter stride length than those in the AD‐MCI group (all *p* < 0.05). AD patients with decreased scores of MNA and MNA^m^ scales, and declined levels of urea nitrogen and vitamin B_12_ in blood had reduced gait speed and gait cadence, and prolonged step length time and stride length time, whereas homocysteine showed the almost opposite results (all *p* < 0.05). In the AD‐MCI group, the score of scale was negatively correlated with the coefficient of variation (CV) of stride length, and the folic acid level was negatively correlated with the CV of stride length and cadence (all *p* < 0.05).

**Conclusions:**

AD patients at the dementia stage had worse nutritional status and gait performance than those at the MCI stage, which was associated with worse global cognition and activities of daily living. Poorer nutritional status was associated with higher gait variability in patients at the MCI stage and with poorer gait performance in patients at the dementia stage. Early identification and intervention of patients with nutritional risk or malnutrition may improve gait performance, thus reducing the risk of falling and cognitive decline, as well as the mortality.

## Graphical abstract

Alzheimer's disease (AD) patients in the dementia stage had worse nutritional status and gait performance than those in the MCI stage. Poorer nutritional status was associated with poorer gait performance in AD patients.

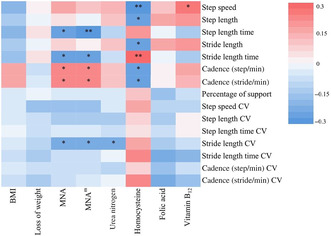

## 1 BACKGROUND

The number of individuals with cognitive impairment is growing as the global aging process continues. Alzheimer's disease (AD) is the most common type of cognitive impairment, characterized by declines of multiple cognitive domains, and varying degrees of neuropsychiatric symptoms and compromised activities of daily living (ADL).^1^ So far, there is no cure for AD. It is therefore crucial to early identify and intervene in the potential risk factors that may contribute to the onset and progression of AD.

According to recommendations from the European Society for Clinical Nutrition and Metabolism (ESPEN), malnutrition and weight loss are the most common nutritional conditions in dementia patients.^2^ In AD patients, malnutrition was associated with accelerated disease progression, and increased morbidity and mortality.^3^, ^4^ Population‐based studies found that weight loss was a predictor of both incident mild cognitive impairment (MCI) and dementia, which might be one of the first signs of cognitive decline.^5^ AD patients with severe dementia had a higher risk of malnutrition and lower body mass index (BMI) than those with MCI and mild dementia.^6^ There are several indicators that can reflect nutritional status, including weight loss, BMI, muscle mass, reduced food intake, burden/inflammation, rating scales, and laboratory variables, such as blood urea nitrogen and B vitamins. Currently, there is a paucity of research that comprehensively assesses the nutritional status of AD patients. Given that nutrition is a modifiable risk factor for AD, one of the aims of this study was to holistically evaluate the nutritional status of AD patients at different stages from multiple perspectives.

Gait is a key indicator of overall health. Neural networks, including cortical, subcortical, and spinal cord structures, are involved in regulating gait control.^7^ AD patients had impaired balance control, slower gait speed, and greater gait variability than individuals with normal cognition.^8^, ^9^ Abnormal gait increases the risk of falling in AD patients, resulting in increased mortality. However, there are still limitations in current research on AD and gait performance. For instance, it is still unclear what the characteristics of gait variability among AD patients at different disease stages. The relationship between different gait parameters and neuropsychiatric symptoms as well as ADL of AD patients remained unknown. Furthermore, the gait parameters included in previous research were not comprehensive enough. This study therefore aimed to comprehensively investigate the relationship between multiple gait parameters and the different stages of AD, as well as its clinical symptoms.

Recently, a cross‐sectional study based on cognitively healthy older adults showed that gait speed was twice as high in the well‐nourished as in the malnourished.^10^ To date, however, the relationships between the nutritional status and gait performance in AD patients have not been studied. The aims of this study were therefore to comprehensively identify the characteristics and correlations of the nutritional status and gait performance in AD patients at different stages of disease.

## 2 METHODS

### 2.1 Subjects

A total of 85 subjects with AD were consecutively enrolled in the cross‐sectional study between September 2021 and November 2022 from the Center for Cognitive Neurology, Department of Neurology, Beijing Tiantan Hospital, Capital Medical University. All subjects were diagnosed with MCI due to AD (AD‐MCI)^11^ or dementia due to AD (AD‐D)^12^ according to the National Institute of Aging and Alzheimer's Association (NIA‐AA) criteria and divided into the AD‐MCI group and the AD‐D group, respectively. AD patients meeting the following criteria were excluded from this study: (1) patients with other neurological diseases that affected cognition, including Parkinson's disease, dementia with Lewy bodies, frontotemporal dementia, corticobasal degeneration, acute cerebrovascular disease involving the cerebral cortex, and acute stroke that was temporally associated with cognitive impairment; (2) patients with diseases affecting gait, including lower extremity fracture, femoral head necrosis, and lower extremity arteriosclerosis obliterans; (3) patients with other conditions leading to malnutrition, including hematological tumors, liver cirrhosis, severe systemic diseases, or subtotal gastrectomy; and (4) patients who were unable to cooperate with all the examinations for various reasons.

### 2.2 Demographic data

The general demographic data, including sex, age, age of onset, education level, marital status, *apolipoprotein E (APOE)* genotype, and several comorbidities, including hypertension, hyperlipidemia, diabetes mellitus, hyperhomocysteinemia, myocardial infarction, atrial fibrillation, cerebral infarction, cerebral hemorrhage, thyroid disease, smoking, and drinking, were collected.

The following five aspects of nutrition‐related demographic data were collected according to the Global Leadership Initiative on Malnutrition (GLIM) criterion^13^:

#### 2.2.1 Non‐volitional weight loss

Non‐volitional weight loss was defined as a self‐reported body weight loss within the past 6 months, which was categorized as <5%, 5%–10%, or >10%.^14^

#### 2.2.2 BMI

BMI was calculated by dividing the measured body weight by the squared height (kg/m^2^) and classified as underweight (BMI <18.5 kg/m^2^), normal weight (BMI 18.5–23.9 kg/m^2^), overweight (BMI 24.0–27.9 kg/m^2^), or obese (BMI ≥28.0 kg/m^2^) according to Chinese adults classification standard.^15^, ^16^

#### 2.2.3 Muscle mass

Circumferences of the arm, waist, hip, and calf were measured to reflect the muscle mass of patients. The waist circumference was measured at the narrowest point of the trunk between the ribs and the upper part of the hip bone. The hip circumference was measured at the widest point of the hip and buttock. The arm circumference was measured at the midpoint between the acromion and olecranon. The calf circumference was measured at the location where the calf was thickest.^17^

#### 2.2.4 Reduced food intake

Reduced food intake referred to a decrease in food intake over the past 3 months due to various reasons, including poor oral health, side effects of medication, depression, dysphagia, gastrointestinal complaints, anorexia, and insufficient nutrition support. It was classified into four categories: no reduction in intake, a 25%–50% reduction, a 50%–75% reduction, or a reduction of ≥75%.^18^

#### 2.2.5 Burden/inflammation

Burden/inflammation was defined as patients with major infections, burns, trauma, closed head injury, fever, negative nitrogen balance, congestive heart failure, chronic obstructive pulmonary disease, rheumatoid arthritis, chronic kidney or liver diseases, or cancer.^13^, ^18^

### 2.3 Clinical symptoms

The global cognition of patients was assessed by the scales of Mini‐Mental State Examination (MMSE)^19^ and the Montreal Cognitive Assessment (MoCA).^20^ Neuropsychiatric symptoms were assessed by the Neuropsychiatric Inventory (NPI) scale.^21^ ADL were assessed by the ADL scale, which includes basic ADL (BADL) and instrumental ADL (IADL).^22^

The nutritional status of patients was assessed by the Mini‐Nutritional Assessment (MNA) scale.^23^ The scoring categorizes subjects: well‐nourished (≥24 points), at risk of malnutrition (17–23.5 points), and malnourished (<17 points). To avoid interference of cognitive performance and depression with nutritional assessment, we also analyzed a modified MNA (MNA^m^) scale, leaving out the question of “neuropsychological problem”.^24^

The detailed description of these scales was provided in the Appendix S1.

### 2.4 Nutrition‐related laboratory variables

The venous blood samples of AD patients were collected from the median elbow under fasting condition the next morning after admission, and then sent to the clinical laboratory of Beijing Tiantan Hospital.

We collected a variety of nutrition‐related laboratory variables in blood to assess the nutritional status of patients, including hemoglobin A1c, fasting blood glucose, hemoglobin, urea nitrogen, creatinine, albumin, prealbumin, homocysteine, folic acid, and vitamin B_12_, in serum.

### 2.5 Gait parameters

The Codamotion 3D motion capture system (Charnwood Dynamics, Ltd., United Kingdom) was used to collect the bilateral gait parameters of patients by active infrared capture.

To record steady‐state walking patterns, subjects were instructed to start walking approximately 1 m before entering the infrared capture range. A successful trial is defined as the acquisition of more than three consecutive complete cycles of stereo‐motion data, and each task requires the acquisition of at least six successful trials.

The gait parameters, including speed (m/s), step length (m), step length time (s), stride length (m), stride length time (s), cadence (steps/min), cadence (strides/min), and percentage of support (%), were collected. Cadence (steps/min) is defined as the number of steps per minute, and cadence (strides/min) is defined as the number of strides per minute. The variability of each gait parameter is represented by the coefficient of variation (CV): standard deviation (SD)/mean × 100.^25^

### 2.6 Statistical analysis

Statistical analyses were performed by SPSS Statistics 25.0 (IBM Corporation, New York, USA). Statistical significance was defined as a two‐sided *p* < 0.05.

Data were tested for normal distribution using the Kolmogorov–Smirnov test. Clinical characteristics, nutritional status, and gait performance were compared between the AD‐MCI and the AD‐D groups. Continuous variables conforming to normal distribution were presented as means ± SD and compared by two‐tailed *t* test, non‐normal distributed variables were presented as median (quartile) and compared by non‐parametric test, and categorical variables were presented as number (percentage) and compared by chi‐squared test. Binary logistic regression analyses were performed to assess the association of nutritional status and gait performance with dementia, with Model 1 was adjusted for age and gender, and Model 2 was further adjusted for age of onset, educational level, and the scores of NPI and ADL scales based on Model 1. Pearson correlation analysis and Spearman correlation analysis were performed to evaluate the correlations between nutritional status and gait performance in the total patients, the AD‐MCI group, and the AD‐D group, respectively, and the results were presented by heat maps.

## 3 RESULTS

**FIGURE 1:**
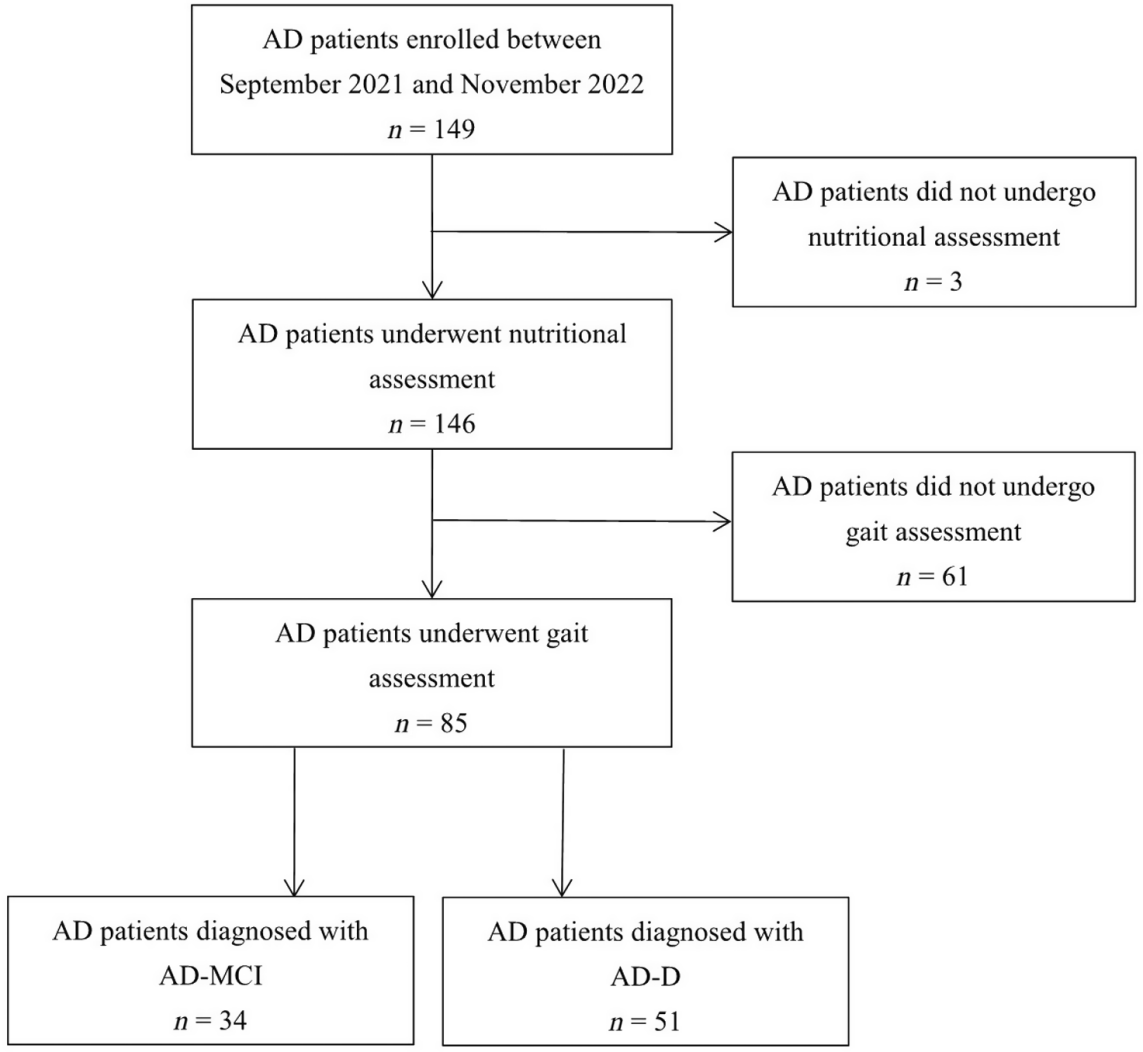
Study flow chart. AD, Alzheimer's disease; MCI, mild cognitive impairment.

A total of 149 patients diagnosed with AD were enrolled consecutively from September 2021 to November 2022, of which three patients who did not undergo nutritional assessment and 61 patients who did not undergo gait assessment were excluded. Finally, 85 patients with AD were enrolled in this study, with 34 cases in the AD‐MCI group and 51 cases in the AD‐D group (Figure 1).

### 3.1 Clinical characteristics of AD patients

The clinical characteristics of AD patients were shown in Table 1. It was showed that 58.82% of patients were females, the average age was 64.13 ± 10.26 years old, the average age of onset was 59.54 ± 11.92 years old, and 32.90% of cases carried *APOE* ε4 allele. There were no statistically significant differences in all demographic variables between the AD‐MCI and the AD‐D groups.

**TABLE 1:** Clinical characteristics of the AD‐MCI and AD‐D groups.

	Total (*n* = 85)	AD‐MCI group (*n* = 34)	AD‐D group (*n* = 51)	*p*
Female (*n*, %)	50 (58.82)	24 (70.59)	26 (50.98)	0.072
Age (years, mean ± SD)	64.13 ± 10.26	63.35 ± 10.69	64.65 ± 10.05	0.577
Age of onset (years, mean ± SD)	59.54 ± 11.92	59.48 ± 10.92	59.58 ± 12.67	0.971
Education level (years, mean ± SD)	11.11 ± 4.97	12.08 ± 4.70	10.46 ± 5.09	0.150
Marital status				
Married (*n*, %)	76 (89.41)	28 (82.35)	48 (94.12)	0.096
Never married (*n*, %)	2 (2.35)	2 (5.88)	0 (0.00)	
Divorced or widowed (*n*, %)	7 (8.24)	4 (11.76)	3 (5.88)	
*APOE* ε4 allele carrier (*n*, %)	28 (32.90)	10 (29.40)	18 (35.30)	0.572
Hypertension (*n*, %)	42 (49.41)	16 (47.06)	26 (50.98)	0.723
Hyperlipidemia (*n*, %)	19 (22.35)	10 (29.41)	9 (17.65)	0.202
Diabetes mellitus (*n*, %)	13 (15.29)	7 (20.59)	6 (11.76)	0.268
Hyperhomocysteinemia (*n*, %)	1 (1.18)	0 (0.00)	1 (1.96)	0.414
Myocardial infarction (*n*, %)	1 (1.18)	1 (2.94)	0 (0.00)	0.400
Atrial fibrillation (*n*, %)	2 (2.35)	2 (5.88)	0 (0.00)	0.151
Smoking (*n*, %)	19 (22.35)	5 (14.71)	14 (27.45)	0.167
Drinking (*n*, %)	16 (18.82)	4 (11.76)	12 (23.53)	0.174
Body weight (kg, mean ± SD)	23.74 ± 3.77	24.43 ± 4.05	23.28 ± 3.54	0.172
Body weight loss within the past 6 months				
<5% (*n*, %)	78 (91.76)	31 (91.18)	47 (92.16)	0.884
5–10% (*n*, %)	7 (8.24)	3 (8.82)	4 (7.84)	
>10% (*n*, %)	0 (0.00)	0 (0.00)	0 (0.00)	
BMI (kg/m^2^, mean ± SD)	23.74 ± 3.77	24.43 ± 4.05	23.28 ± 3.54	
Underweight (*n*, %)	3 (3.53)	0 (0.00)	3 (5.88)	0.172
Normal weight (*n*, %)	44 (51.76)	17 (50.00)	27 (52.94)	
Overweight (*n*, %)	28 (32.94)	12 (35.29)	16 (31.37)	
Obese (*n*, %)	10 (11.76)	5 (14.71)	5 (9.80)	
Circumference				
Waist circumference (cm, mean ± SD)	88.03 ± 11.27	92.04 ± 14.67	85.74 ± 8.34	0.124
Hip circumference (cm, mean ± SD)	100.89 ± 11.02	102.71 ± 10.93	99.83 ± 11.42	0.595
Arm circumference (cm, mean ± SD)	26.88 ± 3.80	27.05 ± 3.06	26.77 ± 4.27	0.777
Calf circumference (cm, mean ± SD)	35.04 ± 4.68	34.90 ± 3.56	35.14 ± 5.35	0.843
Disease burden/inflammation (*n*, %)	4 (4.71)	2 (5.88)	2 (3.92)	0.686
Reduced food intake within the past 3 months				
None (*n*, %)	72 (84.71)	30 (88.24)	44 (86.27)	0.704
25%–50% (*n*, %)	7 (8.24)	2 (5.88)	5 (9.80)	
50%–75% (*n*, %)	3 (3.53)	1 (2.94)	2 (3.92)	
≥75% (*n*, %)	3 (3.53)	1 (2.94)	0 (0.00)	
Global cognition				
MMSE (points, mean ± SD)	16.83 ± 7.75	24.50 ± 2.88	11.62 ± 5.25	<0.001**
MoCA (points, mean ± SD)	12.83 ± 7.13	19.41 ± 4.97	8.26 ± 4.35	<0.001**
Neuropsychiatric symptoms				
NPI [points, median (quartile)]	3.00 (0.00, 9.50)	1.00 (1.00, 3.25)	6.00 (1.00, 15.00)	0.004**
ADL				
ADL [points, median (quartile)]	22.00 (20.00, 26.00)	20.00 (20.00, 21.00)	24.00 (21.00, 32.00)	<0.001**
Nutrition assessment scales				
MNA (points, mean ± SD)	22.85 ± 2.68	24.15 ± 2.36	21.98 ± 2.54	<0.001**
MNA^m^ (points, mean ± SD)	21.74 ± 2.54	22.67 ± 2.41	21.12 ± 2.45	0.006**
MNA category				
Well‐nourished (*n*, %)	35 (41.18)	20 (58.82)	15 (29.41)	0.003**
At risk of malnutrition (*n*, %)	48 (56.47)	14 (41.18)	34 (66.67)	
Malnourished (*n*, %)	2 (2.35)	0 (0.00)	2 (3.92)	
Nutrition‐related laboratory variables in blood				
Hemoglobin A1c (%, mean ± SD)	6.05 ± 0.75	5.91 ± 0.62	6.13 ± 0.82	0.189
FBG (mmol/L, mean ± SD)	133.86 ± 13.32	134.15 ± 13.36	133.67 ± 13.42	0.872
Hemoglobin (g/L, mean ± SD)	5.16 ± 1.17	4.94 ± 0.95	5.30 ± 1.28	0.154
Urea nitrogen (mmol/L, mean ± SD)	5.58 ± 1.26	6.08 ± 1.15	5.25 ± 1.23	0.003**
Creatinine (μmol/L, mean ± SD)	56.91 ± 9.84	56.54 ± 8.49	57.17 ± 10.75	0.779
Total calcium (mmol/L, mean ± SD)	2.31 ± 0.09	2.31 ± 0.07	2.31 ± 0.10	0.692
Total protein (mmol/L, mean ± SD)	64.26 ± 4.69	63.94 ± 4.35	64.48 ± 4.94	0.609
Albumin (g/L, mean ± SD)	39.29 ± 3.01	39.37 ± 2.31	39.23 ± 3.43	0.843
Prealbumin (g/L, mean ± SD)	244.27 ± 35.17	240.32 ± 27.75	246.81 ± 39.30	0.427
Triglyceride [mmol/L, median (quartile)]	1.04 (0.76, 1.45)	1.07 (0.83, 1.53)	1.02 (0.71, 1.40)	0.477
Total cholesterol (mmol/L, mean ± SD)	4.51 ± 0.93	4.54 ± 1.08	4.50 ± 0.82	0.846
HDLC (mmol/L, mean ± SD)	1.42 ± 0.32	1.40 ± 0.37	1.44 ± 0.28	0.644
LDLC (mmol/L, mean ± SD)	2.71 ± 0.84	2.72 ± 0.96	2.70 ± 0.76	0.917
Apolipoprotein A1 (g/L, mean ± SD)	1.43 ± 0.23	1.43 ± 0.23	1.43 ± 0.23	0.969
Apolipoprotein B (g/L, mean ± SD)	0.82 ± 0.20	0.82 ± 0.23	0.81 ± 0.18	0.746
Homocysteine (μmol/L, mean ± SD)	11.98 ± 2.90	10.64 ± 2.41	12.90 ± 2.87	<0.001**
Folic acid [ng/mL, median (quartile)]	6.00 (4.82, 9.22)	8.13 (6.00, 10.91)	5.18 (3.87, 8.13)	0.001**
Vitamin B_12_ [pg/mL, median (quartile)]	362.00 (273.00, 490.00)	476.00 (352.00, 570.50)	320.50 (261.00, 428.75)	0.001**
Ferritin [ng/mL, median (quartile)]	91.75 (58.35, 132.30)	91.40 (68.40, 114.10)	92.10 (56.10, 143.10)	0.801

*Note*: Data were presented as number (percentage), means ± SD or median (quartile).

Abbreviations: AD, Alzheimer's disease; ADL, activities of daily living; APOE, Apolipoprotein E; BMI, body mass index; FBG; fasting blood glucose; HDLC, high‐density lipoprotein cholesterol; LDLC, low‐density lipoprotein cholesterol; MCI, mild cognitive impairment; MMSE, Mini‐Mental State Examination; MNA, Mini‐Nutritional Assessment; MoCA, Montreal Cognitive Assessment; NPI, Neuropsychiatric Inventory.

** *p* < 0.01.

#### 3.1.1 Global cognition, neuropsychiatric symptoms, and ADL of AD patients

The scores of MMSE and MoCA scales were lower (all *p* < 0.001), and the scores of NPI scale (*p* = 0.004) and ADL scale (*p* < 0.001) were higher in the AD‐D group than that in the AD‐MCI group (Table 2).

**TABLE 2:** Association of nutritional status and gait performance with dementia in AD patients.

	Crude		Model 1		Model 2	
	OR (95% CI)	*p*	OR (95% CI)	*p*	OR (95% CI)	*p*
Nutrition scales						
MNA	0.69 (0.55, 0.86)	<0.001**	0.67 (0.53, 0.85)	0.001**	0.71 (0.52, 0.97)	0.033*
MNA^a^	0.76 (0.62, 0.93)	0.009**	0.75 (0.60, 0.94)	0.012*	0.74 (0.55, 0.98)	0.038*
Nutrition‐related laboratory variables in blood						
Urea nitrogen	0.56 (0.37, 0.84)	0.005**	0.51 (0.33, 0.80)	0.003**	0.28 (0.12, 0.67)	0.004**
Homocysteine	1.43 (1.15, 1.78)	0.001**	1.45 (1.15, 1.84)	0.002**	1.50 (1.09, 2.08)	0.014*
Folic acid	0.92 (0.84, 1.01)	0.072	0.91 (0.83, 1.00)	0.055	0.82 (0.68, 0.99)	0.037*
Vitamin B_12_	1.00 (0.99, 1.00)	0.005**	1.00 (0.99, 1.00)	0.008**	1.00 (0.99, 1.00)	0.057
Gait performance						
Step speed	0.03 (0.00, 0.39)	0.007**	0.02 (0.00, 0.34)	0.006**	0.02 (0.00, 0.62)	0.017*
Step length	0.00 (0.00, 0.28)	0.017*	0.00 (0.00, 0.10)	0.009**	0.00 (0.00, 0.48)	0.035*
Stride length	0.03 (0.00, 0.56)	0.019*	0.01 (0.00, 0.34)	0.010*	0.01 (0.00, 0.69)	0.035*

*Note*: Model 1 was adjusted for sex and age; Model 2 was adjusted for sex, age, age of onset, educational level, and the scores of NPI and ADL scale.

Abbreviations: AD, Alzheimer's disease; ADL, activities of daily living; MNA, Mini‐Nutritional Assessment; NPI, Neuropsychiatric Inventory.

* *p* < 0.05.

** *p* < 0.01.

#### 3.1.2 The nutritional status of AD patients

Compared to the AD‐MCI group, AD‐D group had lower scores of MNA (*p* < 0.001) and MNA^m^ scales (*p* = 0.006), and had higher proportion of at risk of malnutrition and malnutrition (*p* = 0.003) (Table 1).

As far as nutrition‐related laboratory variables, the levels of urea nitrogen (*p* = 0.003), folic acid (*p* = 0.001), and vitamin B_12_ (*p* = 0.001) in the AD‐D group were all lower than those in the AD‐MCI group, and the homocysteine level in the AD‐D group was higher than that in the AD‐MCI group (*p* < 0.001) (Table 1).

Binary logistic regression analyses showed that lower scores of MNA [adjusted OR 0.71 (0.52, 0.97), *p* = 0.033] and MNA^m^ scales [adjusted OR 0.74 (0.55, 0.98), *p* = 0.038], lower levels of urea nitrogen [adjusted OR 0.28 (0.12, 0.67), *p* = 0.004] and folic acid [adjusted OR 0.82 (0.68, 0.99), *p* = 0.037], and higher homocysteine level [adjusted OR 1.50 (1.09, 2.08), *p* = 0.014] were related to AD‐D after adjusting for sex, age, age of onset, educational level, and the scores of NPI and ADL scales (Table 2).

We further analyzed the relationship of the nutritional status with global cognition, neuropsychiatric symptoms, and ADL scale in AD patients. The results showed that the scores of MNA and MNA^m^ scales, and the levels of folic acid and vitamin B_12_ were positively correlated with the scores of MMSE scale, MoCA scale, and negatively correlated with the score of ADL scale, while homocysteine level was negatively correlated with the scores of MMSE and MoCA scales, and positively correlated with the ADL score (all *p* < 0.05). The score of MNA scale was also negatively correlated with the score of NPI scale (*r* = −0.331, *p* = 0.002) (Table S1).

#### 3.1.3 Gait assessment of AD patients

**FIGURE 2:**
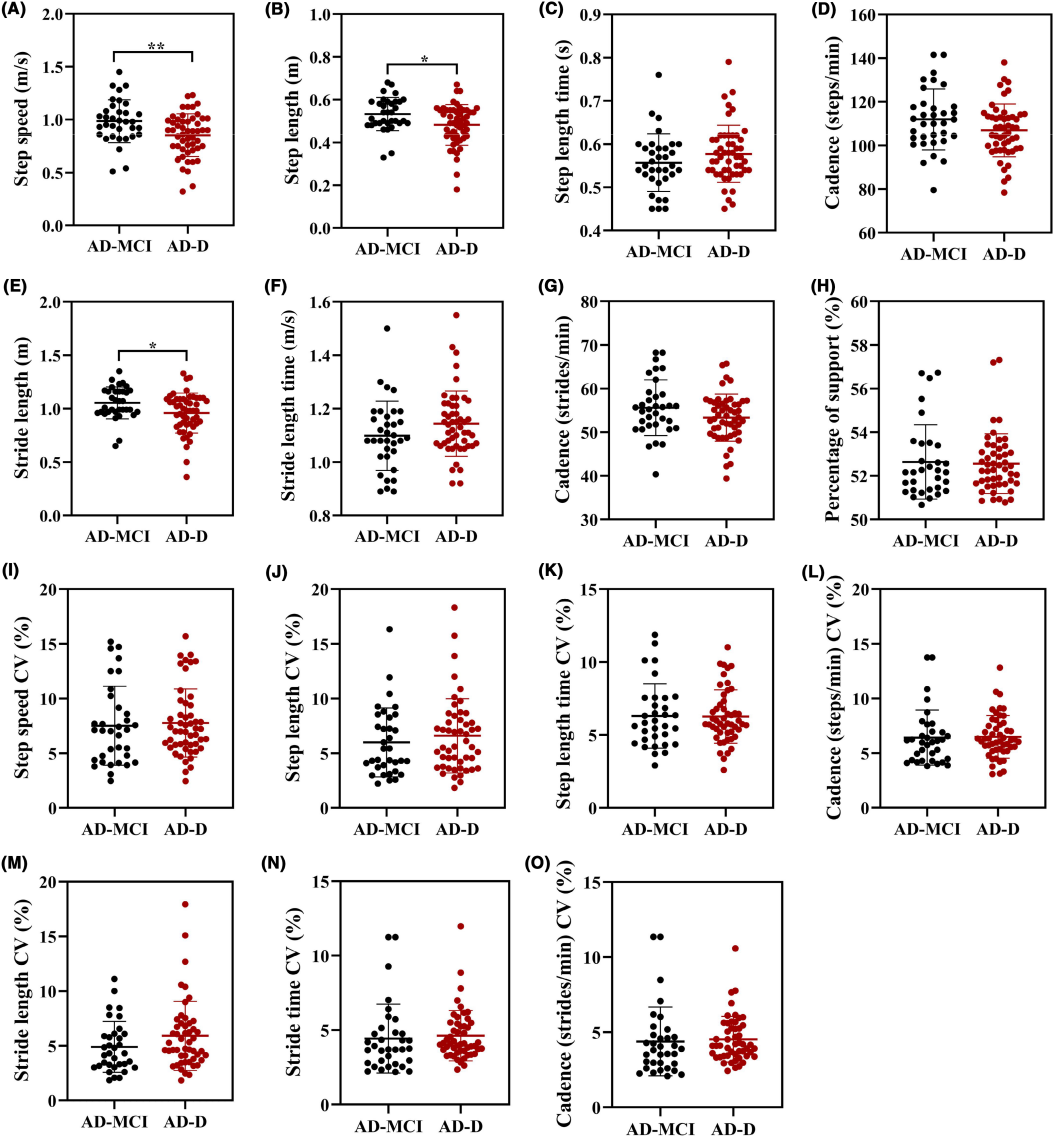
Comparison of Gait performance between the AD‐MCI and AD‐D groups. Comparisons of step speed (A), step length (B), step length time (C), cadence (steps/min) (D), stride length (E), stride length time (F), cadence (strides/min) (G), percentage of support (H), and CV of A‐G (I–O) between the groups of AD‐MCI and AD‐D. AD, Alzheimer's disease; CV, coefficient of variation; MCI, mild cognitive impairment. *p < 0.05, **p < 0.01.

Figure 2 showed the differences in the gait performance between the AD‐MCI and AD‐D groups. Patients in the AD‐D group had slower step speed (*p* = 0.004), shorter step length (*p* = 0.012), and shorter stride length (*p* = 0.014) than those in the AD‐MCI group. The variability of these gait parameters did not differ between the two groups.

Binary logistic regression analysis showed that patients with slower step speed [adjusted OR 0.02 (0.00, 0.62), *p* = 0.017], shorter step length [adjusted OR 0.00 (0.00, 0.48), *p* = 0.035], and shorter stride length [adjusted OR 0.01 (0.00, 0.69), *p* = 0.035] were associated with dementia after adjusting for sex, age, age of onset, educational level, and the scores of NPI and ADL scales (Table 2).

In addition, step speed, step length, stride length, and cadence (steps/min) were positively associated with the scores of MMSE and MoCA scales, and negatively associated with the score of ADL scale (all *p* < 0.05) (Table S1).

### 3.2 Association between nutritional status and gait performance of AD patients

**FIGURE 3:**
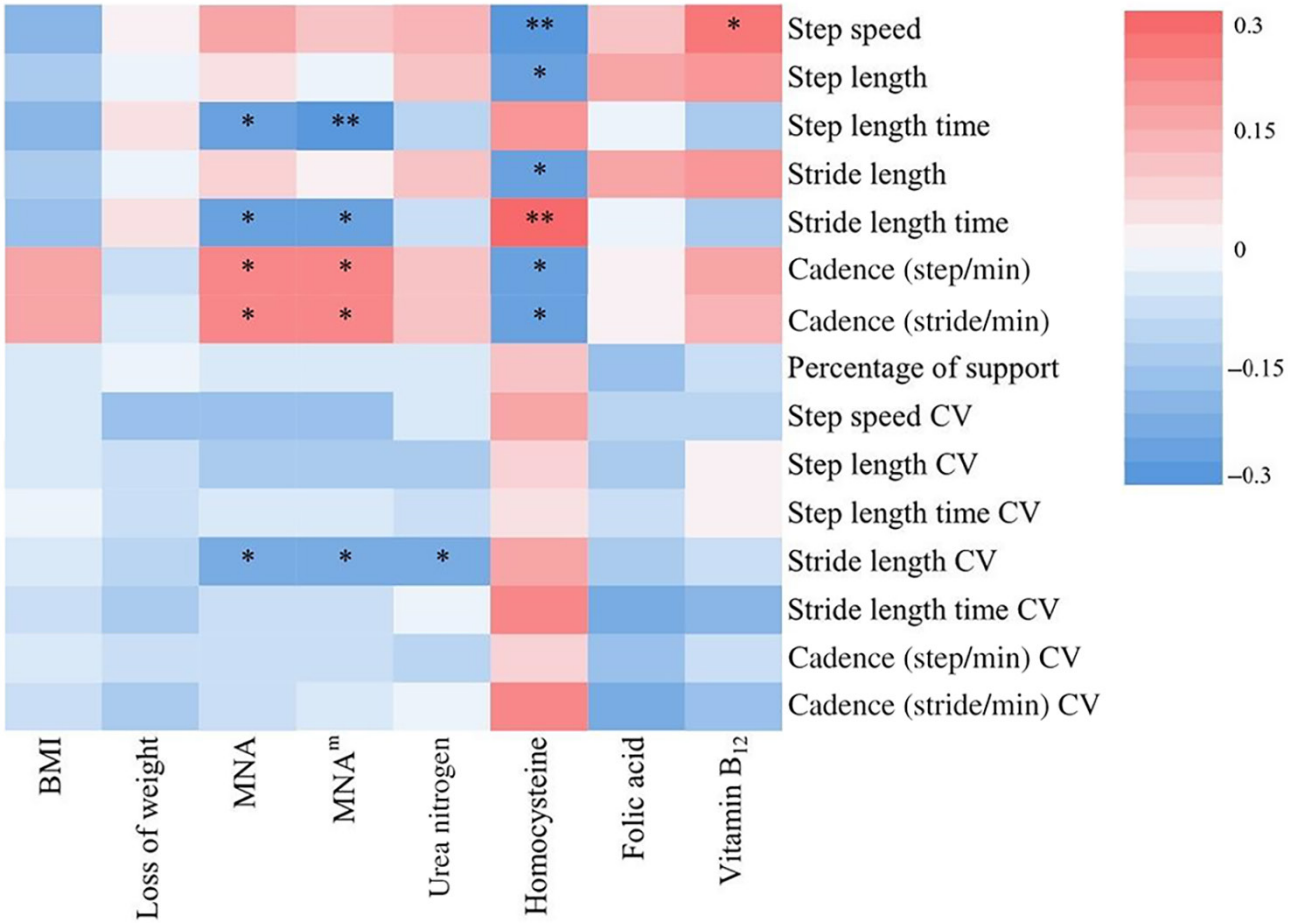
Heat map of correlations between the nutritional status and gait performance in patients with AD. BMI, body mass index; CV, coefficient of variation; MNA, Mini‐Nutritional Assessment. *p < 0.05, **p < 0.01.

The correlations between the nutritional status and gait performance in AD patients were shown in Figure 3.

The score of MNA scale was negatively correlated with step length time (*r* = −0.28, *p* = 0.011), stride length time (*r* = −0.23, *p* = 0.038), and stride length CV (*r* = −0.22, *p* = 0.043), and positively correlated with cadence (steps/min) (*r* = 0.26, *p* = 0.020) and cadence (strides/min) (*r* = 0.23, *p* = 0.036). The score of MNA^m^ scale was similarly correlated with step length time (*r* = −0.30, *p* = 0.006), stride length time (*r* = −0.25, *p* = 0.023), stride length CV (*r* = −0.23, *p* = 0.037), cadence (steps/min) (*r* = 0.26, *p* = 0.018), and cadence (strides/min) (*r* = 0.24, *p* = 0.027).

In the nutrition‐related laboratory variables, homocysteine level was negatively correlated with step speed (*r* = −0.31, *p* = 0.005), step length (*r* = −0.24, *p* = 0.030), stride length (*r* = −0.23, *p* = 0.037), cadence (steps/min) (*r* = −0.24, *p* = 0.034), and cadence (strides/min) (*r* = −0.28, *p* = 0.011), and was positively correlated with stride length time (*r* = 0.29, *p* = 0.009). Blood Vitamin B_12_ level was positively correlated with step speed (*r* = 0.24, *p* = 0.036). Urea nitrogen level was negatively correlated with stride length CV (*r* = −0.24, *p* = 0.029).

**FIGURE 4:**
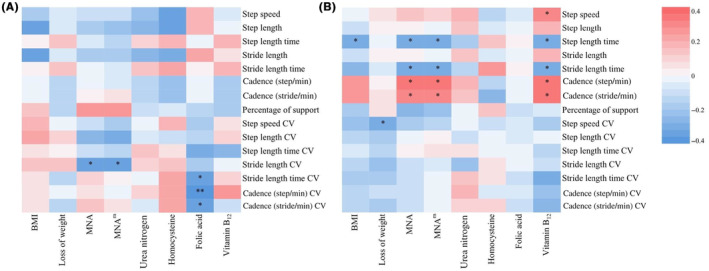
Heat maps of correlations between the nutritional status and gait performance in the AD‐MCI and AD‐D groups. BMI, body mass index; CV, coefficient of variation; MNA, Mini‐Nutritional Assessment. *p < 0.05, **p < 0.01.

Furthermore, analyses were conducted to explore the nutritional status and gait performance in the AD‐MCI and AD‐D groups (Figure 4). In the AD‐MCI group, the nutritional status was related to gait variability. The scores of MNA (*r* = −0.36, *p* = 0.040) and MNA^m^ scores (*r* = −0.39, *p* = 0.025) were all negatively correlated with stride length CV. Folic acid level was negatively correlated with stride length time CV (*r* = −0.39, *p* = 0.037), cadence (steps/min) (*r* = −0.48, *p* = 0.009), and cadence (strides/min) (*r* = −0.38, *p* = 0.045). In the AD‐D group, nutrition‐related variables were significantly correlated with gait parameters. BMI was negatively correlated with step length time (*r* = −0.30, *p* = 0.031). Loss of weight was negatively correlated with step speed CV (*r* = −0.30, *p* = 0.033). The score of MNA scale was negatively correlated with step length time (*r* = −0.35, *p* = 0.013) and stride length time (*r* = −0.29, *p* = 0.044), and positively correlated with cadence (steps/min) (*r* = 0.33, *p* = 0.020) and cadence (strides/min) (*r* = 0.29, *p* = 0.042). Furthermore, vitamin B_12_ level was positively correlated with step speed (*r* = 0.32, *p* = 0.032), cadence (steps/min) (*r* = 0.35, *p* = 0.017), and cadence (strides/min) (*r* = 0.31, *p* = 0.035), and negatively correlated with step length time (*r* = −0.32, *p* = 0.028) and stride length time (*r* = 0.31, *p* = 0.033).

We further explored the differences in gait performance between the stages of MCI and dementia by dividing the patients into the well‐nourished and undernourished risk/malnourished groups according to the score of the MNA scale. In the well‐nourished subgroup, there were no statistical differences in gait parameters and their variability between the stages of MCI and dementia (Figure S1). Nevertheless, in the undernourished risk/malnourished group, step speed (*p* = 0.007), step length (*p* = 0.027), and stride length (*p* = 0.034) in the AD‐MCI group were all significantly lower than that in the AD‐D group (Figure S2).

## 4 DISCUSSION

To our knowledge, this is the first study to elucidate the associations between nutritional status and gait performance in AD patients at different stages. We found that: (1) AD‐D patients had significantly worse nutritional status and gait performance than AD‐MCI patients, which were all significantly related to the worse cognition; (2) nutritional status of patients was markedly and negatively associated with gait variability in the MCI stage, and was remarkably and negatively associated with their gait performance in the dementia stage; (3) in patients with undernourished risk/malnourished, AD‐D patients had worse gait performance than AD‐MCI patients, which was however not found in the well‐nourished patients.

### 4.1 The nutritional status of AD patients was related to disease severity

As weight loss and malnutrition are common problems in AD patients as well as predictors of disease progression and mortality, the nutritional status of individual patient needs to be seriously taken into consideration.^2^, ^3^, ^4^ We found that lower score of MNA scale predicted the occurrence of dementia in AD patients. To improve the reliability of the results, we adopted the revised MNA (MNA^m^) scale and adjusted for sex, age, age of onset, educational level, the score of NPI scale, and the score of ADL scale, and still found a significant and negative association between score of MNA scale and dementia.

The lower scores of MNA and MNA^m^ scales were associated with the lower scores of MMSE and MoCA scales. So far, the factors of malnutrition in AD are not fully clear. It was suggested that the factors that affected food intake, such as olfactory dysfunction, taste disorders, and compromised appetite were the primary reasons for malnutrition in the early stages of AD.^26^, ^27^, ^28^ As the disease progressed, the chronic inflammatory response was gradually increased, leading to excessive protein and energy consumption as the primary cause of malnutrition in the middle and late stages of AD.^29^, ^30^ In addition, eating disorder, dysphagia, and neuropsychiatric symptoms at the later stage of AD exacerbated malnutrition.^31^

In addition, we analyzed the relationship between nutrition‐related laboratory variables and dementia, and found that lower levels of blood urea nitrogen, folic acid, and vitamin B_12_, and higher homocysteine level had good predictive value for dementia. Blood urea nitrogen is the main end product of protein metabolism and one of the indicators reflecting the nutritional status of the body, which was influenced by multiple factors, including protein intake, dehydration, and gastrointestinal bleeding.^32^ When insufficient protein intake or excessive consumption, blood urea nitrogen level will decline. Up to now, almost no literature has reported the relationship between blood urea nitrogen and AD. In this study, the results showed that blood urea nitrogen level in AD‐D patients was significantly lower than that in AD‐MCI patients, and it is reasonable to speculate that this may be related to malnutrition caused by excessive protein and energy consumption at the dementia stage.^29^

B vitamins, including vitamin B_12_ and folic acid, are mainly derived from food intake and are indispensable substances for the metabolism of sugars, fats, and proteins in the human body. These are the components of mono‐carbon metabolism and essential for maintaining cellular methylation capacity.^33^ Multiple studies suggested that low levels of plasma B vitamins in AD patients led to not only the disturbed metabolism of homocysteine but also the impaired release of docosahexaenoic acid and choline into blood, which resulted in increased homocysteine and decreased docosahexaenoic acid and choline, and thus impaired synaptic synthesis and cognition.^34^, ^35^, ^36^

Homocysteine, a kind of sulfur‐containing amino acid in the human body, is either metabolized to cysteine by the transsulfuration pathway or converted back to methionine through the remethylation pathway by using cofactors like folic acid and vitamin B_12_. It was recognized as proatherogenic and prothrombotic, and accepted in many studies as an independent risk for cardiovascular and cerebrovascular diseases.^37^ In addition, homocysteine was reported to be associated with dementia, such as AD‐D, due to vascular damage and oxidative stress.^38^, ^39^

Abnormal nutrition‐related variables are modifiable risk factors for AD; hence, it is important to reveal whether there is a relationship between the levels of above variables and dementia. In this study, AD patients in the dementia stage exhibited lower levels of folic acid and vitamin B_12_. Furthermore, the increased homocysteine and decreased folic acid and vitamin B_12_ were associated with worse global cognition and ADL. These results could potentially be attributed to decreased food intake and increased protein and energy consumptions, which were common in the stage of dementia. The reduced folic acid and vitamin B_12_ in turn impaired homocysteine synthesis, resulting in higher homocysteine level during the dementia stage.

### 4.2 The gait performance of AD patients was related to disease severity

Walking is a complex process related to cognitive ability as well as motor function, and it is sensitive to the loss of a higher level of cognitive control.^7^, ^40^ Previous studies found that patients with cognitive impairment had worse gait performance than those with normal cognition, and poor gait performance acted as a factor predicting progression from MCI to dementia.^9^, ^41^, ^42^ In this study, we comprehensively analyzed the relationship between multiple gait parameters and different stages of AD, and found that slower step speed, shorter step length, and stride length significantly predicted dementia and were associated with worse global cognition and ADL.

Altered gait variability is a common feature of several neurodegenerative diseases, including AD, and its increase reflected worse gait rhythm and was associated with the increased risk of falling, and a harbinger of cognitive decline and disease severity.^42^, ^43^ A previous study found increased gait variability in AD patients compared to cognitively normal individuals, and might distinguish MCI from cognitively normal individuals.^44^ However, the underlying mechanisms of altered gait variability in AD patients are poorly understood. In this study, gait variability between AD‐MCI and AD‐D patients was not different and the association of gait variability with cognition, neuropsychiatric symptoms, and ADL was not found. These results suggested that gait variability might be more useful in distinguishing dementia from non‐dementia than AD at different stages.

### 4.3 The associations between nutritional status and gait performance in AD patients

This study for the first time found a significant correlation between nutritional status and gait performance in AD patients. Patients with lower scores of MNA and MNA^m^ scales, and lower levels of urea nitrogen and vitamin B_12_ had slower gait speed and gait cadence, and longer step length time and stride length time. Patients with higher homocysteine level had slower step speed and cadence, shorter step length and stride length, and longer stride length time. Poor nutritional status was associated with poor muscle strength, leading to poorer gait performance.^10^, ^45^

It is worth noting that the correlation between nutrition and gait is more significant at the dementia phase than at the MCI phase. Gait performance was worse, and the risk of falling was higher in the advanced‐stage patients with AD.^9^, ^41^ The above results indicate that improving nutritional status of patients at the last stage has a greater benefit in improving gait performance, and consequently may be more effective in reducing the risk of falling and mortality compared to the patients at the early stage of AD. More interestingly, we found that nutritional status was markedly related to gait variability in patients at the MCI stage, which was revealed by the results that the score of MNA scale was significantly and negatively correlated with stride length CV, and the folic acid level was significantly and negatively correlated with stride length CV and cadence CV. However, the association was not significant in AD patients at the dementia stage, except that weight loss was negatively correlated with step speed CV. Higher gait variability was associated with worse gait rhythm, walking stability, and a higher risk of falling.^42^, ^43^ The above results were critical for early identification of AD patients at high risk of falling and cognitive decline.

In this study, in AD patients with impaired nutrition, patients at the dementia stage had slower step speed, shorter step length, and stride length than those at the MCI stage; however, in the well‐nourished AD patients, this differences in gait performance were not evident between the cases at different stages. We speculate that AD patients with malnutrition may have lower muscle mass and worse muscle strength, which may lead to impaired gait performance. Thus, nutritional status may accelerate the deterioration of gait performance as the disease progresses.^10^, ^45^

## 5 LIMITATION

This study had limitations. First of all, the cross‐sectional nature hampers causal interpretation of our findings, and further longitudinal studies with large samples are needed. Second, the differences of nutritional status and gait performance among the cognitively normal individuals, patients with other cognitive disorders, and AD patients need to be further explored in the future.

## 6 CONCLUSION

AD patients at the dementia stage had both poorer nutritional status and worse gait performance than those at the MCI stage, which were associated with worse global cognition and ADL. Poorer nutritional status was associated with higher gait variability in patients at the MCI stage, and poorer gait performance in patients at the dementia stage. Nutrition is one of the risk factors that can be intervened with, and early identification and intervention of AD patients with nutritional risk or malnutrition may improve gait performance, thus reducing the risk of falling, delaying the progression of AD symptoms, and decreasing the mortality of AD patients.

## AUTHOR CONTRIBUTIONS

Mingyue He contributed to the conception, design, and data statistics of the study and manuscript writing; Tenghong Lian, Peng Guo, Jing Qi, Jinghui Li, Huiying Guan, Dongmei Luo, Zhan Liu, Weijia Zhang, Zijing Zheng, Hao Yue, Jing Li, Wenjing Zhang, Ruidan Wang, and Fan Zhang contributed to the acquisition and collation of data; Yanan Zhang, Yue Huang, and Xiaomin Wang contributed to directing manuscript writing and data statistics; and Wei Zhang contributed to the conception and design of the study and the revision of manuscript writing.

## FUNDING INFORMATION

This research was supported by the National Key Research and Development Program of China (2016YFC1306300, 2016YFC1306000); the National Natural Science Foundation of China (81970992, 81571229, 81071015, 30770745, 82201639); the Capital's Funds for Health Improvement and Research (CFH) (2022‐2‐2048); the Key Technology R&D Program of Beijing Municipal Education Commission (kz201610025030); the Key Project of Natural Science Foundation of Beijing, China (4161004); the Natural Science Foundation of Beijing, China (7082032); the Project of Scientific and Technological Development of Traditional Chinese Medicine in Beijing (JJ2018‐48); the Capital Clinical Characteristic Application Research (Z121107001012161); the High Level Technical Personnel Training Project of Beijing Health System, China (2009‐3‐26); the Project of Beijing Institute for Brain Disorders (BIBD‐PXM2013_014226_07_000084); the Excellent Personnel Training Project of Beijing, China (20071D0300400076); the Project of Construction of Innovative Teams and Teacher Career Development for Universities and Colleges Under Beijing Municipality (IDHT20140514); the Beijing Healthcare Research Project, China (JING‐15‐2); the Basic‐Clinical Research Cooperation Funding of Capital Medical University, China (2015‐JL‐PT‐X04, 10JL49, 14JL15); and the Natural Science Foundation of Capital Medical University, Beijing, China (PYZ2018077).

## CONFLICT OF INTEREST STATEMENT

The authors report no competing interests.

## Supporting information


Appendix S1.

## Data Availability

The data are available from the first author upon reasonable request.
