# Supplementary material for: Association between nutritional status and gait performance in Alzheimer's disease
Source: CNS Neurosci Ther. 2023 Nov 10;30(4):e14502. doi: 10.1111/cns.14502 (PMC11017406; doi:10.1111/cns.14502)
Supplement: Supplementary file 1 — Appendix S1. [file CNS-30-e14502-s001.docx]

**Supplementary material**

**Assessments of clinical symptoms**

*Global cognition:* Global cognition of patients was assessed by the scales of Mini-Mental State Examination (MMSE)[1] and the Montreal Cognitive Assessment (MoCA)[2]. MMSE is a one-page 30-point test. First, orientation to time and place is evaluated. The immediate memory is evaluated by learning trial of three nouns and short-term memory recall is assessed after evaluating attention and calculation ability with a serial subtraction task. Language is assessed by repeating a sentence, following the instructions written on the card, performing a sentence, and saying a complete and meaningful sentence. Finally, visuospatial ability is assessed by copying an overlapping pentagon. Patients with illiteracy, primary education, or more than a junior education were identified as dementia when the MMSE score was below 17, 20 or 24 points, respectively. MoCA is a one-page 30-point test administered in 10 minutes. The short-term memory recall task involves two learning trials of five nouns and delayed recall after approximately 5 minutes. Visuospatial abilities are assessed using a clock-drawing task and a three-dimensional cube copy. Multiple aspects of executive functions are assessed using an alternation task adapted from the Trail Making B task, a phonemic fluency task, and a two-item verbal abstraction task. Attention, concentration, and working memory are evaluated using a sustained attention task (target detection using tapping), a serial subtraction task, and digits forward and backward. Language is assessed using a three-item confrontation naming task with low-familiarity animals (lion, camel, rhinoceros), repetition of two syntactically complex sentences, and the aforementioned fluency task. Finally, orientation to time and place is evaluated. The MoCA score ≤ 26 indicated cognitive impairment and 1 point was added if the educational level of an individual was less than 12 years. The lower the scores of the two scales, the poorer the global cognitive function.

*Neuropsychiatric symptoms:* Neuropsychiatric symptoms were assessed by the Neuropsychiatric Inventory (NPI) scale[3]. NPI consists of 10 items, including delusions, hallucinations, agitation/aggression, depression/dysthymia, anxiety, euphoria, apathy, disinhibition, irritability/emotional instability, and abnormal motor behavior, and 2 autonomic symptoms, including sleep/nocturnal behavior and appetite/eating disorders. Each item was assessed by its frequency (1-4 points) and severity (1-3 points), frequency and severity scores were multiplied to obtain a score (0-12 points) for each item, and the total scores were summed to obtain a total NPI score (0-144 points). The scores from caregivers represents the NPI-Caregiver Burden, which reflects caregiver distress caused by neuropsychiatric symptoms in AD patients. The higher the scale score, the more severe the overall neuropsychiatric symptoms.

*Activities of Daily Living (ADL):* ADL scale were used to assess the activities of daily living[4]. The basic ADL (BADL) and instrumental ADL (IADL) scales were included. BADL assesses whether patients can catch a bus, go to a place near their home, cook, do their own household chores, take their medication, eat, dress and brush their hair and teeth. The IADL assesses whether patients can wash their own clothes, walk around a flat room, walk up and downstairs, get in and out of bed, sit or stand, carry water for cooking or bathing, take a bath, cut toenails, shop, go to the bathroom regularly, make phone calls, handle their own money and stay home alone. Patients were assessed on how much they did it themselves and how much their families helped. The higher the score, the worse the daily living ability.

*Nutritional status:* The nutritional status of patients was assessed by the Mini-Nutritional Assessment (MNA) scale[5]. This scale consists of 18 questions and rapid measurement items that can be performed in less than 15 minutes. The scale involves 4 parts: anthropometric assessment (BMI, arm and calf circumferences and weight loss), general assessment (6 questions related to lifestyle, medication and mobility), dietary assessment (8 questions related to number of meals, food and fluid intake and autonomy of feeding), and subjective assessment (self-perception of health and nutrition). The scoring categorizes subjects in the following manner: well-nourished (≥ 24 points), at risk of malnutrition (17-23.5 points) and malnourished (< 17 points).

**References**

1. Folstein MF, Folstein SE, McHugh PR. "Mini-mental state". A practical method for grading the cognitive state of patients for the clinician. J Psychiatr Res. 1975, 12(3):189-198.

2. Nasreddine ZS, Phillips NA, Bédirian V, Charbonneau S, Whitehead V, Collin I, et al. The Montreal Cognitive Assessment, MoCA: a brief screening tool for mild cognitive impairment. J Am Geriatr Soc. 2005, 53(4):695-699.

3. Cummings JL, Mega M, Gray K, Rosenberg-Thompson S, Carusi DA, Gornbein J. The Neuropsychiatric Inventory: comprehensive assessment of psychopathology in dementia. Neurology. 1994, 44(12):2308-2314.

4. Mathuranath PS, George A, Cherian PJ, Mathew R, Sarma PS. Instrumental activities of daily living scale for dementia screening in elderly people. Int Psychogeriatr. 2005, 17(3):461-474.

5. Guigoz Y, Vellas B, Garry PJ. Assessing the nutritional status of the elderly: The Mini Nutritional Assessment as part of the geriatric evaluation. Nutr Rev. 1996, 54(1 Pt 2):S59-65.

**Supplementary Table**

**Supplementary Table 1 Correlations between global cognition,** **neuropsychiatric symptoms, ADL, nutritional status and gait performance in AD patients**

|  | **MMSE** |  | **MoCA** |  | **NPI** |  | **ADL** |  |
| --- | --- | --- | --- | --- | --- | --- | --- | --- |
|  | r | *P* | r | *P* | r | *P* | r | *P* |
| BMI | 0.085 | 0.440 | 0.057 | 0.608 | -0.090 | 0.413 | 0.094 | 0.392 |
| Loss of weight | 0.027 | 0.807 | 0.094 | 0.397 | -0.107 | 0.328 | -0.081 | 0.461 |
| MNA | 0.375 | < 0.001** | 0.294 | 0.008** | -0.331 | 0.002** | -0.198 | 0.074 |
| MNA^m^ | 0.246 | 0.026* | 0.157 | 0.161 | -0.264 | 0.017 | -0.065 | 0.559 |
| Blood urea nitrogen | 0.178 | 0.110 | 0.149 | 0.182 | -0.111 | 0.316 | -0.068 | 0.541 |
| Homocystein | -0.331 | 0.003** | -0.347 | 0.002** | -0.033 | 0.771 | 0.220 | 0.049* |
| Folic acid | 0.363 | < 0.001** | 0.329 | 0.004** | -0.131 | 0.261 | -0.328 | 0.004** |
| Vitamin B_12_ | 0.462 | < 0.001** | 0.475 | < 0.001** | -0.216 | 0.063 | -0.421 | < 0.001** |
| Step speed | 0.303 | 0.005** | 0.354 | < 0.001** | -0.098 | 0.373 | -0.289 | 0.007** |
| Step length | 0.261 | 0.017* | 0.310 | 0.004** | 0.003 | 0.980 | -0.287 | 0.008** |
| Step length time | -0.201 | 0.066 | -0.208 | 0.057 | 0.208 | 0.056 | 0.116 | 0.290 |
| Stride length | 0.257 | 0.018* | 0.302 | 0.005** | -0.002 | 0.988 | -0.292 | 0.007** |
| Stride length time | -0.197 | 0.072 | -0.220 | 0.044* | 0.204 | 0.061 | 0.094 | 0.390 |
| Cadence (Steps/min) | 0.230 | 0.035* | 0.244 | 0.025* | -0.187 | 0.086 | -0.127 | 0.246 |
| Cadence (Strides/min) | 0.204 | 0.063 | 0.228 | 0.037* | -0.201 | 0.066 | -0.090 | 0.412 |
| Percentage of support | 0.033 | 0.766 | 0.107 | 0.333 | -0.004 | 0.970 | -0.021 | 0.846 |
| Step speed CV | -0.074 | 0.505 | 0.001 | 0.991 | 0.192 | 0.079 | 0.064 | 0.561 |
| Step length CV | -0.084 | 0.446 | -0.033 | 0.764 | 0.156 | 0.155 | 0.050 | 0.652 |
| Step length time CV | -0.024 | 0.828 | -0.046 | 0.679 | -0.022 | 0.841 | 0.012 | 0.911 |
| Stride length CV | -0.178 | 0.106 | -0.121 | 0.274 | 0.213 | 0.051 | 0.075 | 0.494 |
| Stride length time CV | -0.176 | 0.108 | -0.147 | 0.182 | 0.060 | 0.584 | 0.151 | 0.167 |
| Cadence (Steps/min) CV | -0.096 | 0.387 | -0.095 | 0.392 | -0.005 | 0.962 | 0.113 | 0.303 |
| Cadence (Strides/min) CV | -0.148 | 0.178 | -0.128 | 0.246 | 0.067 | 0.541 | 0.123 | 0.264 |

Abbreviation: AD, Alzheimer’s disease; MMSE, Mini-Mental State Examination; MoCA, Montreal Cognitive Assessment; NPI, Neuropsychiatric Inventory; ADL, activities of daily living; BMI, BMI, body mass index; MNA, Mini-nutritional Assessment; CV, coefficient of variation. **P* < 0.05, ***P* < 0.01.

**Supplementary Figures**

**
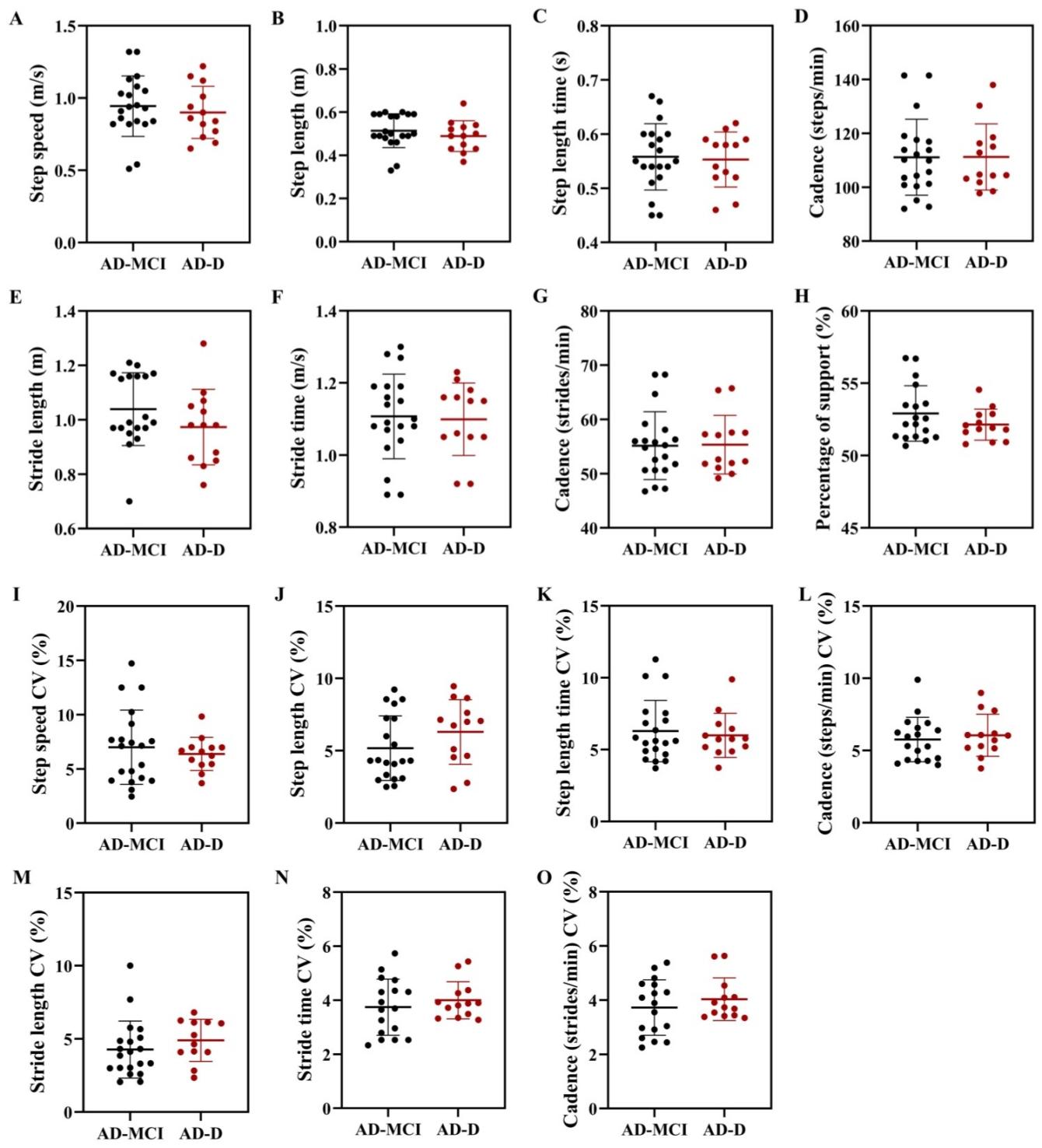
**

**Supplementary Figure 1 Gait performance between the AD-MCI and AD-D groups in well-nourished patients.** Comparisons of step speed (A), step length (B), step length time (C), cadence(steps/min) (D), stride length (E), stride time (F), cadence(strides/min) (G), percentage of support (H) and their CV (I-O) between the groups of AD-MCI and AD-D in well-nourished patients. Abbreviation: AD, Alzheimer’s disease; MCI, mild cognitive impairment; CV, coefficient of variation.


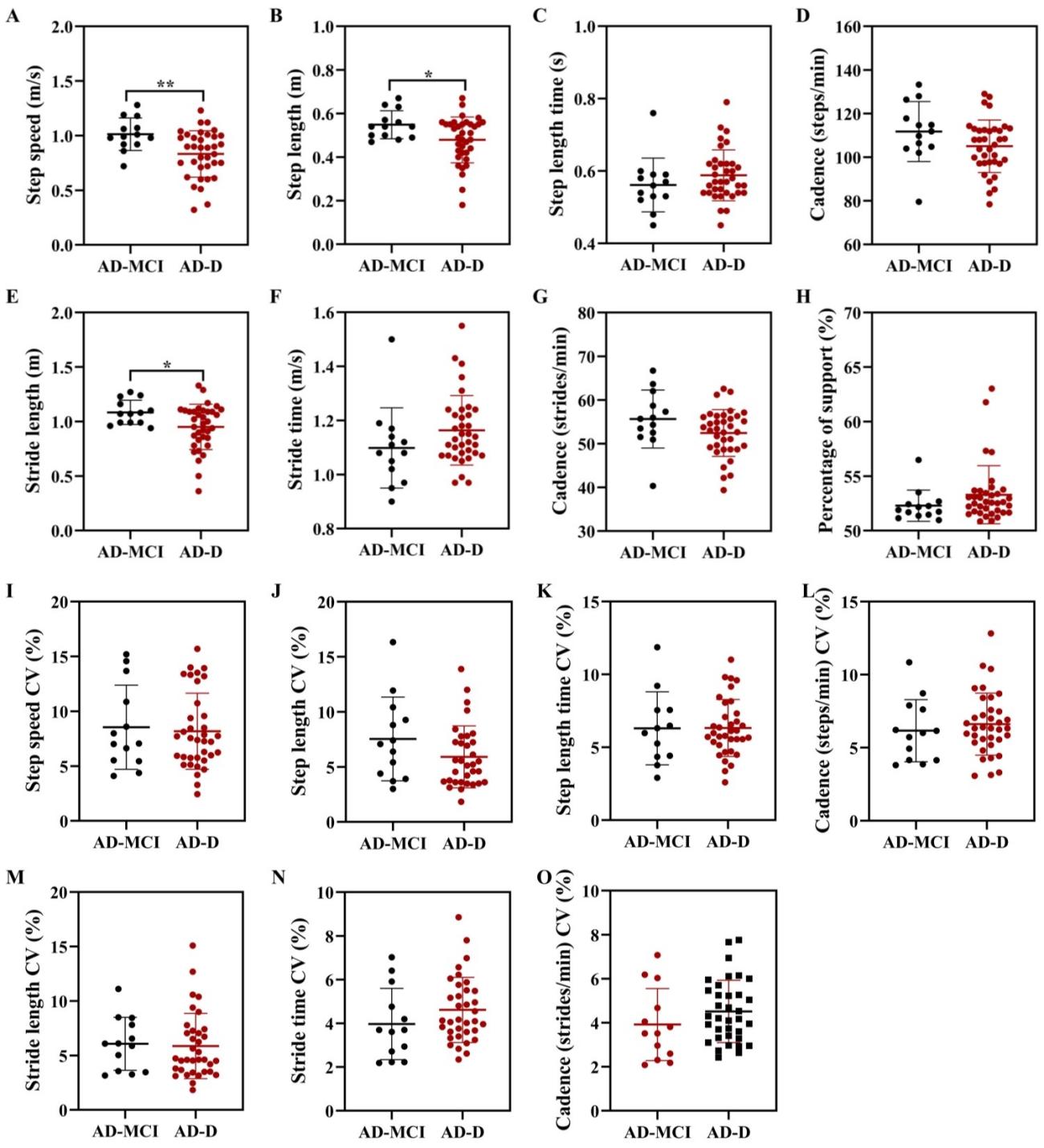


**Supplementary Figure 2 Gait performance between the AD-MCI and AD-D groups in the undernourished risk/malnourished patients.** Comparisons of step speed (A), step length (B), step length time (C), cadence(steps/min) (D), stride length (E), stride time (F), cadence(strides/min) (G), percentage of support (H) and their CV (I-O) between the groups of AD-MCI and AD-D in the undernourished risk/malnourished patients. Abbreviation: AD, Alzheimer’s disease; MCI, mild cognitive impairment; CV, coefficient of variation. **P* < 0.05, ***P* < 0.01.
